# Neuroleptic Malignant Syndrome: A Case of Unknown Causation and Unique Clinical Course

**DOI:** 10.7759/cureus.14113

**Published:** 2021-03-25

**Authors:** Brooke J Olson, Mohan S Dhariwal

**Affiliations:** 1 Internal Medicine, Medical College of Wisconsin, Milwaukee, USA

**Keywords:** neuroleptic malignant syndrome, antipsychotic medications, neuroleptics, adverse drug reaction, neuropharmacology, neuroleptic medications, psychotic disorder, dopamine

## Abstract

Neuroleptic malignant syndrome (NMS) is a rare, potentially lethal syndrome known to be related to the initiation of dopamine antagonist medications or rapid withdrawal of dopaminergic medications. It is a diagnosis of exclusion with a known sequela of symptoms, but not all patients experience these characteristic symptoms making it difficult at times to diagnose and treat. Herein, we present a unique case of NMS with unclear etiology and a unique clinical course. Our case report also raises the question of whether or not adjusting doses of previously prescribed neuroleptic medications can provoke NMS, providing valuable information for providers treating these complex patients.

## Introduction

Neuroleptic malignant syndrome (NMS) is a rare, potentially lethal syndrome known to be related to the initiation of dopamine antagonist medications or rapid withdrawal of dopaminergic medications. The incidence of this uncommon condition ranges from 0.02% to 3% of patients taking antipsychotic medications, most commonly affecting young men given high-dose antipsychotics [1]. While easily recognizable when all classic symptoms are present, the heterogeneity of its clinical course makes this syndrome difficult to identify, commonly left as a diagnosis of exclusion [2]. With this case report, we present a diagnosis of NMS with unclear etiology and unique clinical course. 

## Case presentation

A 33-year-old female with a past medical history of mild cognitive impairment, intermittent explosive disorder, impulse control disorder, tardive dyskinesia, attention deficit hyperactivity disorder (ADHD), hypothyroidism, and non-epileptiform seizures presented to the emergency department with symptoms of altered mental status. According to the patient’s group home, she had been having slower speech, increased drowsiness, and drooling which began four days prior to her presentation. At this point in time, caretakers were still administering her daily medications which included lurasidone, diazepam, and haloperidol for agitation, valbenazine for tardive dyskinesia, trazodone for sleep, and clonidine for ADHD. Her symptoms had progressed to increased lethargy, bowel and bladder incontinence, mutism, and eventually, the patient became unresponsive, prompting her visit to the ED. 

Notably, the patient recently had an appointment with her psychiatrist approximately two weeks prior to her presentation. At this appointment, multiple changes were made to her medication regimen. Her haloperidol schedule was adjusted from 5 mg twice a day to 5 mg nightly with 5 mg as needed dosing for agitation. After speaking with the patient’s caretakers at her group home, it was unclear how much haloperidol they were administering in addition to her scheduled dosing. There was suspicion that her caretakers had been administering increased dosages of haloperidol than before due to the patient’s history of combative behavior. The patient was also taking benztropine 1 mg nightly for extrapyramidal symptoms which were discontinued at this visit. She had been tapering this medication from 2 mg to 1 mg with the intent to stop taking the medication entirely to reduce polypharmacy. At this appointment, benztropine was discontinued due to no evidence of movement disorder and also already having valbenazine prescribed for tardive dyskinesia.

In the ED, infectious and acute trauma workups were all negative. Physical examination in the ED showed a lethargic patient with cogwheel rigidity in the bilateral upper extremities and minimal withdrawal to deep painful stimuli. The patient’s symptoms improved slightly with 1 mg of lorazepam. NMS, malignant catatonia, or meningitis were suspected resulting in admission for further workup. On admission, the patient was lethargic, unable to speak, unable to move her extremities, and was diffusely hyperreflexic with hypertonia. Haloperidol levels were obtained, and results came back at 5.4 ng/mL. The therapeutic level of haloperidol ranges from 5 ng/mL to 20 ng/mL. Creatinine kinase levels were obtained and were significantly elevated with a maximum of 3328 units/L. She was afebrile on admission with a temperature of 98°F but experienced undulating fevers throughout the entirety of her stay with a maximum temperature of 100.4°F on two separate occasions. Other vital signs were within normal limits during her admission. A lumbar puncture and brain MRI were also obtained which were both negative. Our patient also experienced episodes of seizure-like activity with emesis. These episodes were presumed to be related to her history of non-epileptiform seizures. Continuous electroencephalogram showed no seizure activity. 

Neurology and psychiatry teams were consulted and therapy with intravenous (IV) dantrolene 150 mg was recommended. The patient has eventually transitioned to IV dantrolene 100 mg every six hours and oral amantadine 100 mg every eight hours. The day after the initiation of dantrolene therapy, hypertonia and hyperreflexia improved. Approximately four days after initiation of dantrolene therapy, the patient’s mental status significantly improved starting with increasing speech. She worked with physical therapy and occupational therapy during her stay for weakness. Dantrolene taper was initiated and the patient's strength significantly improved. By the time of discharge, the patient was near her baseline level of activity. The patient’s stay lasted 16 days total. 

## Discussion

The pathophysiology of NMS is complex and poorly understood. The connection between dopamine antagonists and NMS is well-known, leaving dopamine blockade paramount to many theories on the etiology of NMS. Specifically, blockade of the D2 dopamine receptors located in the hypothalamus and brainstem have been proposed [2]. Therefore, any agent that blocks the D2 dopaminergic pathway can cause NMS, including metoclopramide and prochlorperazine. Unique dopamine pathway blockade has been linked to the defining symptoms of NMS. Disruption of the regulatory systems in the brainstem has been linked to the systemic hypermetabolic syndrome, central dopamine blockade has been linked to hyperthermia and signs of dysautonomia, and nigrostriatal dopamine blockade has been linked to rigidity and tremor [3-6]. Musculoskeletal fiber toxicity has also been suggested as a possible etiology of NMS given the clinical similarities between NMS and malignant hyperthermia [7]. Patients diagnosed with NMS have also been studied for findings of genetic predisposition. Detection of a specific allele expressed in the dopamine D2 receptor gene was found to be over-represented in NMS patients [8].

The onset and duration of NMS are highly variable. Literature reports that in patients diagnosed with NMS, up to 16% developed symptoms within 24 hours of initiation of an antipsychotic agent, 66% by one week, and 96% within 30 days [9,10]. The DSM-V criteria for diagnosing NMS include major criteria, which must be present, including exposure to a dopamine-blocking agent, severe muscle rigidity, and fever. It also includes other criteria, of which at least two must be met. Other criteria include diaphoresis, dysphagia, tremor, incontinence, mutism, altered mental status, etc. Other findings include elevated creatinine kinase, urinary incontinence, and diaphoresis [11]. The clinical course varies from patient to patient, although a common sequence of symptom development in NMS patients has been identified. Studies have shown that NMS patients typically first present with changes in mental status, followed by muscular rigidity, hyperthermia, and autonomic dysfunction [3]. Treatment of NMS includes stopping the suspected causative agent, supportive care, and medical therapy. Medical therapy includes bromocriptine, dantrolene, and lorazepam [1]. Most cases of NMS resolve within two weeks of therapy with a mean reported recovery time of seven to eleven days [1,9,12].

We present a unique case of NMS of unclear etiology. Our patient’s presenting symptoms fit the defined sequelae of NMS with the exclusion of all other possible differential diagnoses. Her presentation of altered mental status which then progressed to muscle rigidity and hyperthermia is classically seen in NMS, although she did not present with any outright symptoms of autonomic dysfunction. Our patient’s fever history did differ from typical NMS presentation in that she fluctuated from afebrile to febrile states throughout the entirety of her stay, whereas fever in NMS is usually very high with no major fluctuations [7]. Her condition did significantly improve with the administration of dantrolene, consistent with NMS.

What makes our case unusual is that our patient had been taking haloperidol for years prior to this event and had no addition of new neuroleptic agents for months. Her haloperidol regimen was changed near the onset of her symptoms with the suspicion that the patient had been receiving higher dosing than prescribed, but a serum haloperidol level was obtained with unremarkable results. The half-life of haloperidol in the setting of chronic dosing, as seen in our patient, has been noted to be up to 21 days [13]. Haloperidol is a high potency, a first-generation antipsychotic that has been identified as the causative agent in 44% of all patients with NMS [14]. Our patient was also taking lurasidone, an atypical antipsychotic that also has the potential of causing NMS. There were no recent adjustments made to her dose and she had been tolerating this medication for months, so it was not thought to be the causative agent of our patient’s diagnosis.

It has also been reported that NMS can result from withdrawal from anti-Parkinsonian medications [1]. Specifically, there have been cases in which NMS developed after withdrawal of bromocriptine, carbidopa, and levodopa in patients with Parkinson’s disease [15-17]. In these cases, symptoms of NMS appeared roughly one to two weeks after abrupt discontinuation or alteration of dopaminergic medications. It is thought that the increase in dopaminergic transmission with the use of anti-Parkinsonian drugs may lead to NMS when these medications are discontinued [15]. Our patient was taking anti-Parkinsonian medications for tardive dyskinesia. Benztropine was prescribed for extrapyramidal symptoms, which was discontinued approximately two weeks prior to her presentation. She had also been taking valbenazine for these symptoms. This also could have contributed to our patient’s development of NMS, though it is unlikely because benztropine is an anticholinergic medication that does not interfere with the dopaminergic pathway. While valbenazine is an anti-dopaminergic medication, our patient had been on this medication for months with no alterations in dosing. Therefore, there had been no rapid withdrawal or switching of dopaminergic drugs, another known etiology of NMS.

Our case report raises the question of whether or not the modification of antipsychotic medication dose has the potential to trigger NMS. While it is well known that the introduction of an anti-dopaminergic and rapid withdrawal of dopaminergic medications has the potential of causing NMS, we speculate whether or not seemingly minor alterations in already existing regimens potentiate this lethal syndrome. Literature reports that rapidly increasing doses of antipsychotic medications can be a risk factor for the development of NMS as well as taking multiple antipsychotic medications at one time [18]. Our patient was taking multiple antipsychotic medications but did not have a rapid increase in dosing that we are aware of as her haloperidol levels do not support this. 

## Conclusions

Our case provides insight into the various presentations that can be seen in NMS as well as the various possible etiologies. This case highlights the importance of considering the effects associated with alterations in antipsychotic medication regimens prior to launching into broader workups. Also, the administration of antipsychotic medications should be closely monitored and recorded appropriately as this is extremely important in preventing accidental overdosing or unwanted outcomes. With a greater understanding of the presentation and cause of NMS comes decreased time to recognition and treatment. This will further improve outcomes and mortality rates in patients diagnosed with this life-threatening syndrome.
